# m6A regulators are associated with osteosarcoma metastasis and have prognostic significance: A study based on public databases

**DOI:** 10.1097/MD.0000000000025952

**Published:** 2021-05-21

**Authors:** Wenpeng Zhang, Lina Wang, Ping Zhang, Quanbin Zhang

**Affiliations:** a Department of Orthopaedics, Zibo Central Hospital; b Department of Clinical Laboratory, Zibo Mental Health Center; c Department of Ear Nose Throat, Huantai Branch, Qilu Hospital of Shandong University, Zibo, Shandong, China.

**Keywords:** Gene Expression Omnibus, m6A regulators, metastasis, osteosarcoma, prognosis, the Cancer Genome Atlas

## Abstract

**Background:**

Osteosarcoma represents the most common malignant bone tumor with high metastatic potential and inferior prognosis. RNA methylation (N6-methyladenosine [m6A]) is a prevalent RNA modification that epigenetically influences numerous biological processes including tumorigenesis. This study aims to determine that m6A regulators are significant biomarkers for osteosarcoma, and establish a prognostic model to predict the survival of patients.

**Methods:**

In this study, we comprehensively analyzed the underlying associations between m6A regulators’ mRNA expressions and metastasis as well as prognosis of osteosarcoma patients in the Cancer Genome Atlas. Multivariate Cox-regression analysis was used to screen regulators that were significantly associated with overall survival of osteosarcoma patients. Least absolute shrinkage and selection operator (LASSO) Cox-regression analysis was used for constructing m6A regulator-based osteosarcoma prognostic signature.

**Results:**

Some of the regulators exhibited aberrant mRNA levels between osteosarcoma samples with and without metastasis. Multivariate Cox-regression analysis identified several regulators with potential prognostic significance. A risk score formula consisted of methyltransferase-like 3, YTH domains of *Homo sapiens*, and fat mass and obesity-associated protein was obtained through which patients could be prognostically stratified independently of potential confounding factors. The signature was also significantly associated with the metastatic potential of osteosarcoma. All the analyses could be well reproduced in another independent osteosarcoma cohort from the Gene Expression Omnibus.

**Conclusions:**

In conclusion, this study first revealed potential roles of m6A regulators in osteosarcoma metastasis and prognosis, which should be helpful for its clinical decision-making.

## 1 Introduction

Osteosarcoma is the most common primary malignancy of bone.^[1]^ The 5-year event free survival for patients with localized osteosarcoma is approximately 70% to 80%, however, for patients with metastases and relapses, the survival rate is less than 20%.^[2]^ Current treatment approach of osteosarcoma is surgical treatment for the gross tumors while controlling the metastases with systemic chemotherapy.^[3]^ The development of new treatment strategies has always been at the forefront of osteosarcoma research. Osteosarcoma is extremely heterogeneous in both its origins and manifestations, which is associated with various genomic alterations.^[4]^ Conventional osteosarcoma shows extreme genetic instability, and around 80% of the cases show loss of heterozygosity and genomic instability signatures.^[4]^ Since 2008, new genetic indicators have been identified every year.^[5]^ Unfortunately, despite the progress in this field, the survival rate has not been quite improved, which highlights the need for further exploration.^[6]^

Genome integrity can be affected by various endogenous or exogenous events, and prevention and/or repair of genomic DNA damage can respond to these stressors to maintain cellular survival.^[7]^ The defective component in DNA damage and repair machinery is one of the core factors that influence treatment outcome in osteosarcoma.^[8]^ Previous reports have indicated that RNA-mediated DNA repair system plays a direct and active role in genome modification and remodeling, which may have important implications in gene targeting and gene therapy.^[9,10]^ N6-methyladenosine (m6A) is an RNA modification that can alter RNA structure and regulate RNA stability as well as RNA metabolism.^[11–13]^ Moreover, m6A has been associated with the tumorigenesis and development of several tumor types, such as leukemia, glioblastoma, lung, and liver cancers,^[14,15]^ however, the role of m6A in osteosarcoma remains poorly understood. Recently, m6A RNA methylation is proved to regulate the UV-induced DNA damage response through triggering the recruitment of DNA repair proteins.^[16]^ Thus, we propose that it is significant to clarify the correlation between m6A regulators and the metastasis and prognosis of osteosarcoma.

In this study, the information of 88 osteosarcoma tumor samples was obtained from the Cancer Genome Atlas database. We analyzed the underlying association between the expression of m6A regulators and metastasis as well as prognosis, and identified the key regulators with potential prognostic value, and constructed a prognosis signature based on these regulators. This signature was also tested using the osteosarcoma data from the Gene Expression Omnibus database.

## 2 Materials and methods

### 2.1 Study population

All the subjects in this study were obtained from the publicly accessible sources. Training set was acquired from the Cancer Genome Atlas which consisted of 88 osteosarcoma tumor samples. Three samples were excluded from the training set for lack of complete survival information. Testing set was obtained from the Gene Expression Omnibus with the accession number of GSE21257 which included 53 osteosarcoma tumor samples. All samples of this study were from primary tumors, and the clinicopathological characteristics of osteosarcoma patients in the training and testing sets were shown in Table S1, Supplemental Digital Content. As all the subjects here were obtained from the publicly accessible sources, and no human or animal subjects were included, ethical approval was not necessary for our study.

### 2.2 Expression data

Expression levels of mRNA from osteosarcoma tumor tissues in the training and testing sets were quantified through high-throughput RNA-sequencing and Illumina human-6 v2.0 expression beadchip, respectively. A total of 12 and 9 m6A regulators were detected in the training and testing set, respectively, with 8 overlaps. The mRNA levels of those regulators between osteosarcoma samples with different clinicopathological features were analyzed.

### 2.3 Construction of m6A regulator-based prognostic signature for osteosarcoma

Multivariate Cox-regression analysis was applied to assess the associations between m6A regulators and overall survival (OS) probability of osteosarcoma patients in the training set. Regulators exhibiting significant prognostic relevance were retained and used in LASSO Cox-regression analysis to construct the osteosarcoma prognostic signature through which each sample could be assigned a risk score.

### 2.4 Survival analysis

We used Kaplan–Meier method to estimate osteosarcoma patients’ OS probability. The difference in OS probability between osteosarcoma subgroups was determined by log-rank test. *P*-value < .05 was used as the significant threshold.

### 2.5 Statistical analysis

Comparison of mRNA levels between subgroups of osteosarcoma patients was performed by Wilcoxon rank sum test. Multivariate Cox-regression analysis was used to adjust the effects of confounding factors on OS probability. All the statistical analyses in this study were performed in R version 3.4.1.

## 3 Results

### 3.1 m6A regulators are associated with osteosarcoma metastatic status

**Figure 1:**
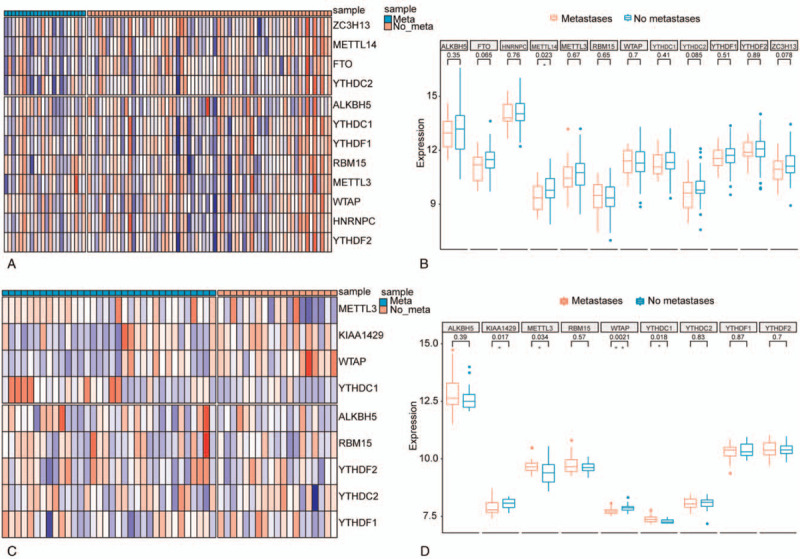
Expression landscape of the 12 m6A regulators in osteosarcoma samples. (A) Heatmap illustrating mRNA expression values of the 12 m6A regulators across the 88 osteosarcoma samples from TCGA (training set). (B) Boxplots illustrating comparison of each m6A regulator's mRNA expression values between osteosarcoma samples with and without metastasis from TCGA (training set). Wilcoxon *P* values were provided above the boxplots. (C) Heatmap illustrating mRNA expression values of the 9 m6A regulators that were profiled by Illumina expression microarray across the 53 osteosarcoma samples from GSE21257 dataset (testing set). (D) Boxplots illustrating the comparison of each m6A regulator's mRNA expression values between osteosarcoma samples with and without metastasis from GSE21257 dataset (testing set). Wilcoxon *P* values were provided above the boxplots. “Meta” represents osteosarcoma samples with metastasis, while “no meta” represents osteosarcoma samples without metastasis; ^∗^ and ^∗∗^ represents *P*-value < .05 and .01, respectively. TCGA = the Cancer Genome Atlas.

The number of m6A regulators identified in both training and testing datasets was shown in Figure S1, Supplemental Digital Content. The mRNA levels of m6A regulators were compared between osteosarcoma samples with and without metastasis in both training (Fig. 1A) and testing (Fig. 1C) sets. As a result, methyltransferase-like 14 (METTL14) exhibited significantly decreased mRNA level in osteosarcoma samples with metastasis compared with those without metastasis in the training set as shown in Figure 1B. Another 2 regulators including fat mass and obesity-associated protein (FTO), and zinc finger CCCH domain-containing protein 13 (ZC3H13) could achieve marginal significance in the training set. Four out of the 9 regulators including protein virilizer homolog (KIAA1429), methyltransferase-like 3 (METTL3), Wilms’ tumor 1-associated protein (WTAP), and YTH domains of *Homo sapiens* (YTHDC1) were significantly differentially expressed in osteosarcoma samples with metastasis compared with those without metastasis in the testing set as shown in Figure 1D.

### 3.2 m6A regulators have prognostic significance

**Figure 2:**
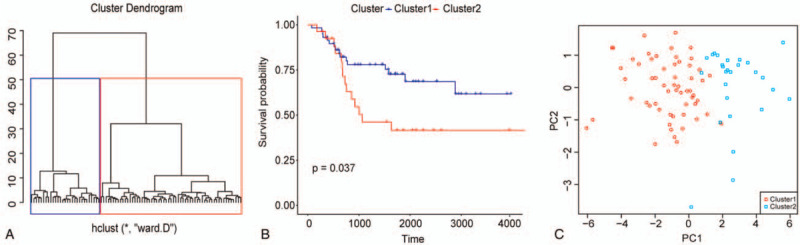
m6A regulators could stratify osteosarcoma patients that were prognostically different. (A) Clustering of osteosarcoma samples from TCGA based on their mRNA expression values of the 12 m6A regulators via K-means method. (B) Kaplan–Meier survival curves of osteosarcoma patients from TCGA stratified by cluster. (C) PCA scatter plot of osteosarcoma samples from TCGA along first 2 principal components. Scatter shapes and colors are used to distinguish samples within different K-means clusters. PCA = principle component analysis, TCGA = the Cancer Genome Atlas.

Osteosarcoma samples in training set were clustered into 2 subgroups via K-means method based on their Euclidean distance that was calculated through the 13 m6A regulators’ mRNA expression values (Fig. 2A). Besides, principle component analysis could definitely separate the samples within the 2 subgroups as shown in Figure 2B, which should indicate the vital roles of the 13 regulators in defining samples. What's more, the 2 groups exhibited significantly different OS probabilities (Fig. 2C), which implied the prognostic significance of these 13 m6A regulators in osteosarcoma.

### 3.3 Prognostic signature

**Figure 3:**
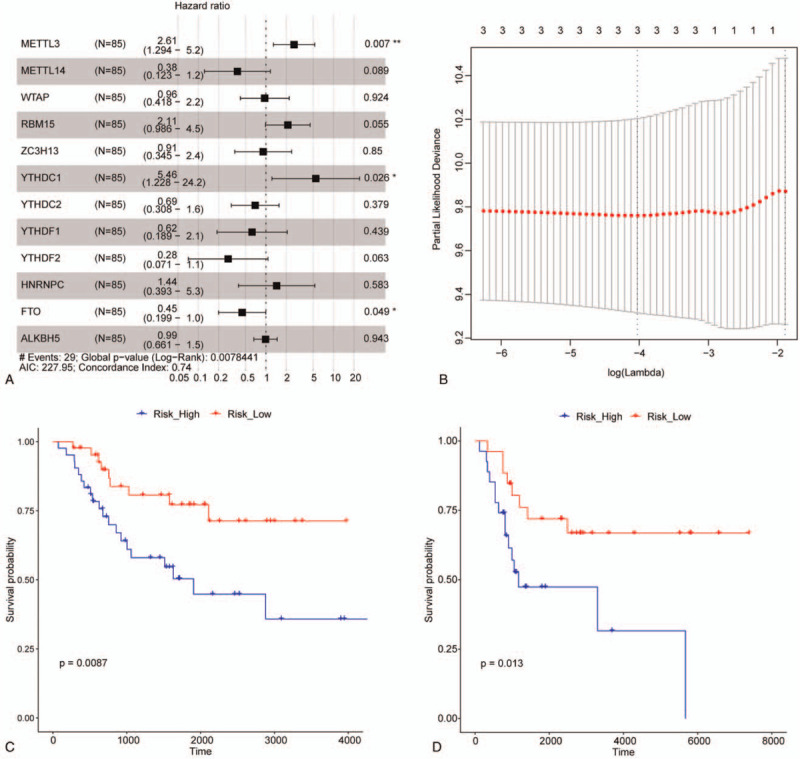
Construction of m6A regulator-based prognostic signature for osteosarcoma. (A) Forest plot of multivariate Cox-regression analysis illustrates associations between each m6A regulator and overall survival of osteosarcoma patients from TCGA. Square data indicates hazard ratio and error bar is 95% confidence interval. ^∗^ and ^∗∗^ represents *P*-value < .05 and .01, respectively. (B) Risk score distribution of osteosarcoma samples in training set. (C) and (D) are the Kaplan–Meier survival curves of osteosarcoma patients from TCGA (training set) and GSE21257 (testing set), respectively. Samples are stratified by the median risk score. TCGA = the Cancer Genome Atlas.

Multivariate Cox-regression analysis identified METTL3, YTHDC1, and FTO as significant osteosarcoma OS probability-associated markers in training set (Fig. 3A). Prognostic signature was built through LASSO Cox-regression analysis based on the mRNA expressions of these 3 significant regulators through which each sample could be assigned a risk score. Figure 3B illustrated distribution of risk score of osteosarcoma samples in training set. Besides, higher risk score was associated with inferior OS probability of osteosarcoma in both training and testing sets as shown in Figure 3C and D.

### 3.4 Clinical relevance of prognostic signature

**Figure 4:**
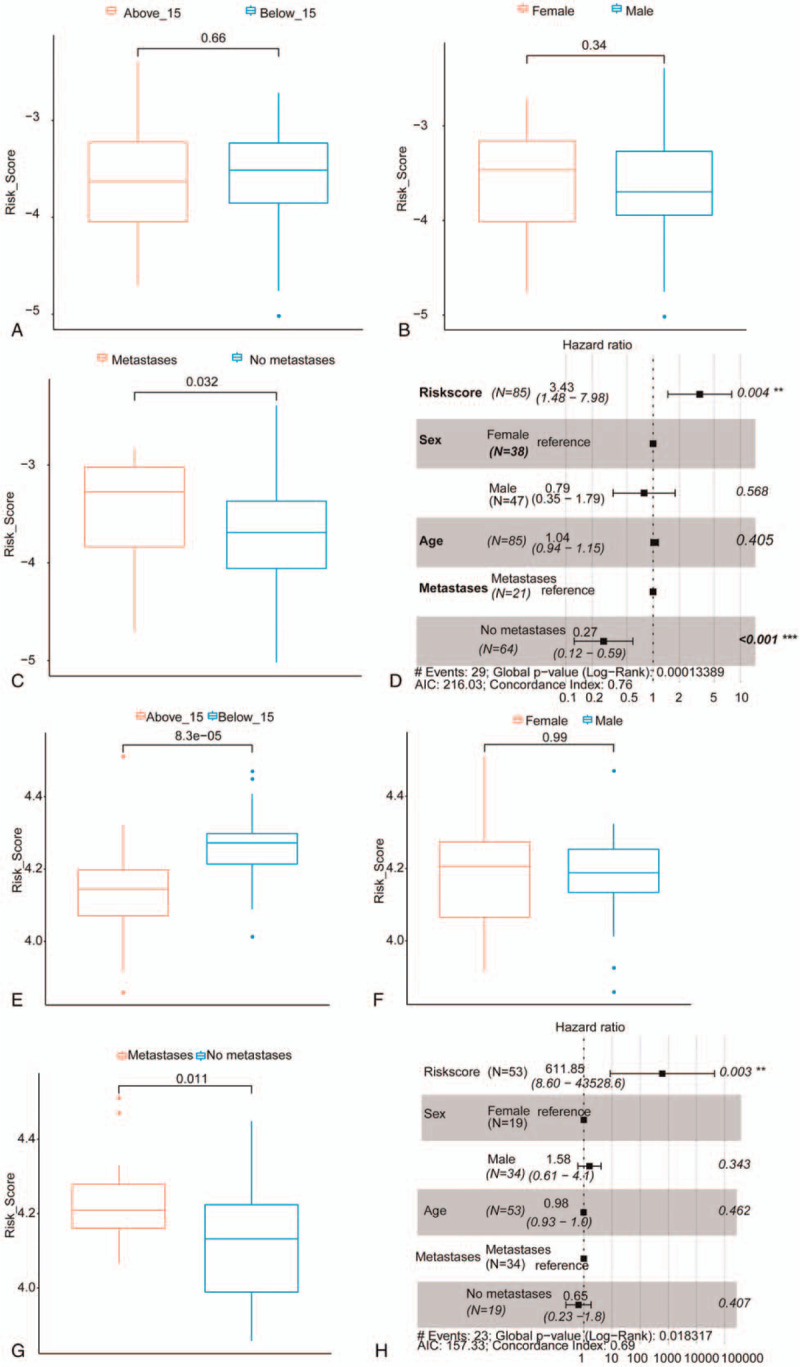
Clinical relevance of risk score. (A–C) Boxplots illustrating comparison of risk score for osteosarcoma samples from TCGA (training set) stratified by their age, gender, and metastatic status. Wilcoxon *P* values are provided above the boxplots. (D) Forest plot of multivariate Cox-regression analysis indicates risk score as an independent prognostic signature for osteosarcoma patients from TCGA (training set) after adjusting for confounding factors including age, gender, and metastatic status. (E–G) Boxplots illustrating comparison of risk score for osteosarcoma samples from GSE21257 dataset (testing set) stratified by their age, gender, and metastatic status. Wilcoxon *P* values are provided above the boxplots. (H) Forest plot of multivariate Cox-regression analysis indicates risk score as an independent prognostic signature for osteosarcoma patients from GSE21257 dataset (testing set) after adjusting for confounding factors including age, gender, and metastatic status. TCGA = the Cancer Genome Atlas.

Here we proposed to explore the potential associations between prognostic signature and common clinicopathological features of osteosarcoma patients including age, gender, and metastatic status. As a result, there was no significant difference in risk score between female and male osteosarcoma samples in both training and testing set (Fig. 4B and F). Strikingly, significant difference in the risk score was observed between osteosarcoma samples that stratified by age of 15 in testing set (Fig. 4E), but not in training set (Fig. 4A). Risk score in osteosarcoma samples with metastasis was significantly higher than that in samples without metastasis in both training and testing sets as shown in Figure 4C and G. What's more, risk score was still an unfavorable factor for the OS probability of osteosarcoma after adjusting for the effects of age, gender, and metastatic status in both training and testing sets (Fig. 4D and H).

## 4 Discussion

In this study, we identified several m6A regulators whose expressions were significantly different between osteosarcoma samples with and without metastasis, including METTL14, FTO, ZC3H13, KIAA1429, METTL3, WTAP, and YTHDC1 (Fig. 1), suggesting that m6A RNA modifications should be involved in the metastasis of osteosarcoma. The m6A regulators can be divided into writers, erasers, and readers by their functions of adding, removing, or recognizing m6A-modification, respectively.^[17]^ The prominent m6A regulators include METTL3, WTAP, RNA binding motif protein 15 (RBM15), ZC3H13, YTHDC1, YTHDC2, YTHDF1, YTHDF2, heterogeneous nuclear ribonucleoprotein C (HNRNPC), FTO, α-ketoglutarate-dependent dioxygenase alkB homolog 5 (ALKBH5), METTL14, and KIAA1429, whose expressions have been studied in gliomas.^[18]^ METTL3, METTL14, and WTAP are the crucial components of the m6A methyltransferase complex.^[19]^ METTL3 methylates target sequence in single strand RNA,^[20,21]^ with a potential function in the regulation of mRNA metabolism.^[22]^ The main function of METTL14 is providing a scaffold for RNA binding, thereby promoting the catalytic activity of METTL3.^[23–25]^ Additionally, METTL14 also plays a key role in various biological processes including embryonic development, gametogenesis, and neurogenesis.^[26–28]^ Another member of m6A methyltransferase complex, WTAP, regulates gene transcription via the interaction with METTL3 and METTL14^[29]^ and involves in the regulation of cell apoptosis.^[22]^ KIAA1429 and ZC3H13 are also required in the complex for m6A methylation.^[30,31]^ KIAA1429 exhibits an essential function in the regulation of methyltransferase complex by recruiting core components of catalyzing,^[30]^ while ZC3H13 participates in the regulation of embryonic stem cell renewal via promoting m6A methylation, which plays an adaptive role between RBM15/Nito and m6A machinery.^[31]^ RBM15 functions in methylation by binding the complex of WTAP-METTL3 and shows a selective activity towards X-inactive specific transcript (XIST).^[32]^ YTHDC1 is one of the m6A readers and regulates splicing as well as nuclear export of mRNA modified by m6A.^[32,33]^ YTHDC2 is larger than other members of YTH family, thus it is endowed diverse functions, such as regulation of RNA binding and structure, and interaction with other complexes.^[34,35]^ An interaction between YTHDF1 and initiation of translating is observed, which improves the translation efficiency.^[36]^ While YTHDF2 could enhance degrading effects of the transcripts which are modified by m6A through recruitment of deadenylase complex.^[37]^ HNRNPC mediates RNA transcripts processing, in which RNA structure is altered by m6A to make RNA transcripts accessible for HNRNPC binding.^[38]^ FTO is a demethylase that demethylates m6A in RNA and DNA,^[39,40]^ and its function is adaptive in various substrates. ALKBH5, localizing to nucleus, is the second identified demethylase. It works in a specific condition in which m6A is the preferential sequence.^[41]^ We performed this study in an attempt to investigate the expression and potential role of these 13 regulators in osteosarcoma. We found that in osteosarcoma samples with metastasis, METTL14 exhibited significantly decreased expression, and WTAP were significantly differentially expressed. The regulation of METTL14 expression on the metastatic behavior of tumor cells varies in different tumors. METTL14 down-regulation would promote the metastatic potential of tumor cells through modulating the primary microRNA 126 process in hepatocellular carcinoma,^[42]^ while it was reported as an oncogenic signal in acute myeloid leukemia.^[43]^ In osteosarcoma, the expression of METTL14 was downregulated in osteosarcoma multidrug-resistant cells as well as tumor-initiating cell,^[44]^ suggesting that it may play a vital role in the emergence and maintaining of osteosarcoma cells.

Through analyzing the prognosis information of osteosarcoma patients, METTL3, YTHDC1, and FTO were identified as significant markers associated with OS probability. METTL3 is a m6A writer and involved in multiple cancers. In hepatoblastoma, METTL3 is associated with cancer development through Wnt/β-catenin signalling pathway and considered as a prognosis predictor. Miao W's group have reported that METTL3 were able to promote osteosarcoma progression by regulating the m6A level of lymphoid enhancer-binding factor 1 (LEF1), and silencing of METTL3 contributed to decrease in m6A methylation and mRNA level of LEF1, thereby suppressing Wnt/β-catenin signaling pathway.^[45]^ Thus it is speculated that METTL3 may associate with osteosarcoma prognosis via Wnt/β-catenin signaling pathway. FTO is a m6A eraser and has been demonstrated to promote acute myeloid leukemia, melanoma, and cervical cancer cells.^[46–48]^ It was reported that, the expression of FTO was significantly decreased in the process of honokiol-induced osteosarcoma cell apoptosis,^[28]^ however, its expression appeared to be independent of the drug-resistance induced by doxorubicin of osteosarcoma cells.^[44]^ In papillary thyroid carcinoma, a prognostic signature including FTO showed good performance in prognosis prediction,^[49]^ but its prognostic value and underlying mechanism in osteosarcoma has not been investigated yet. YTHDC1, a m6A reader, is a member of the YT521-B homology (YTH) domain family, which identifies the m6A group to exercise different downstream effects,^[50,51]^ and was demonstrated to be associated with the progression of prostate cancer.^[52]^ YTHDC1 was proved to be a prognostic factor in ovarian cancer with TP53 mutation,^[53]^ but its prognostic role in osteosarcoma is still unknown. To our knowledge, we are the first to report that osteosarcoma patients could be prognostically stratified using METTL3, YTHDC1, and FTO, which is independent of potential confounding factors including age, gender, and metastatic status. However, the underlying mechanism is still not confirmed and warrants further study.

Due to the complexity of tumor epigenetic regulation, the transfer of m6A biomarkers to the daily clinical practice still faces many challenges, and there are also some limitations in this study. First, we just analyzed the relationship between expression of m6A regulators and OS of osteosarcoma patients due to the limited data. Second, we clustered the osteosarcoma samples into 2 subgroups based on Euclidean distance that was calculated through the expressions of m6A regulators, however, we did not perform in-depth analysis of differences in their biological processes. Based on the results of this study, we would collect clinical samples to further investigate the relationship of m6A regulators with the effect of osteosarcoma chemotherapy, and explore the regulatory mechanisms of the key m6A regulators at the cellular level.

## 5 Conclusion

In this study, we comprehensively analyzed associations between mRNA expressions of m6A regulators with the metastasis and prognosis of osteosarcoma patients, and found that several m6A regulators exhibited aberrant mRNA levels with metastatic and prognostic significance. METTL3, YTHDC1, and FTO were identified as key regulators, and the osteosarcoma prognostic signature based on these m6A regulator could prognostically stratify the patients independently of potential confounding factors. This study highlighted the important role of RNA modification in screening of new therapeutic targets and prognosis prediction of osteosarcoma.

## Author contributions

**Conceptualization:** Quanbin Zhang.

**Data curation:** Wenpeng Zhang, Quanbin Zhang.

**Formal analysis:** Quanbin Zhang.

**Methodology:** Wenpeng Zhang, Quanbin Zhang.

**Project administration:** Quanbin Zhang.

**Software:** Wenpeng Zhang, Lina Wang, Ping Zhang, Quanbin Zhang.

**Supervision:** Wenpeng Zhang, Lina Wang, Ping Zhang, Quanbin Zhang.

**Validation:** Wenpeng Zhang, Lina Wang, Ping Zhang, Quanbin Zhang.

**Visualization:** Wenpeng Zhang, Lina Wang, Ping Zhang, Quanbin Zhang.

**Writing – original draft:** Wenpeng Zhang, Quanbin Zhang.

**Writing – review & editing:** Wenpeng Zhang, Quanbin Zhang.

## Supplementary Material

Supplemental Digital Content

## Supplementary Material

Supplemental Digital Content
