# Supplementary figures and images for: m6A regulators are associated with osteosarcoma metastasis and have prognostic significance: A study based on public databases
Source: Medicine (Baltimore). 2021 May 21;100(20):e25952. doi: 10.1097/MD.0000000000025952 (PMC8137066; doi:10.1097/MD.0000000000025952)

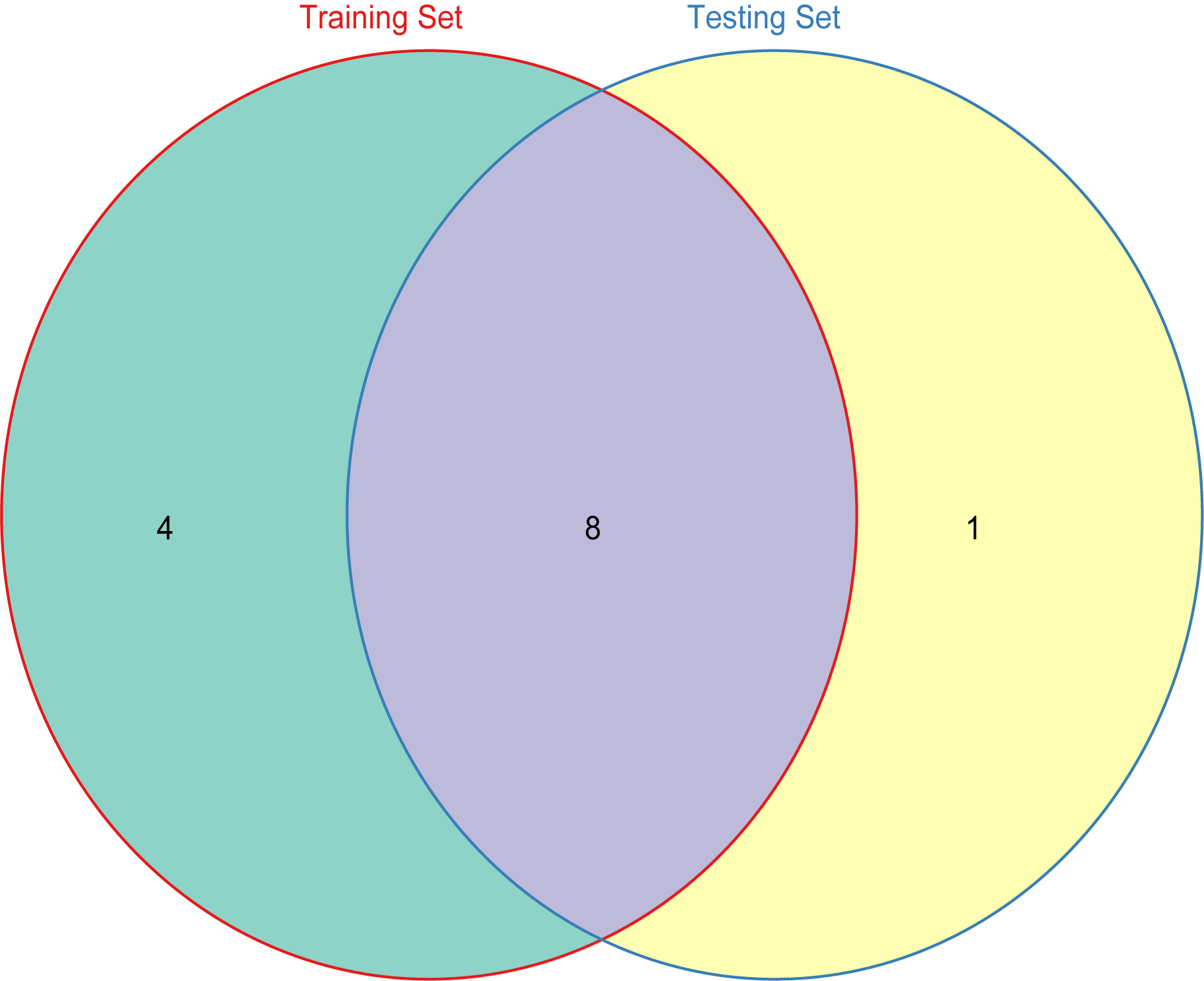

Supplement: Supplemental Digital Content [file medi-100-e25952-s002.tif]
